# Dual-layer dual-energy CT for improving differential diagnosis of squamous cell carcinoma from adenocarcinoma at gastroesophageal junction

**DOI:** 10.3389/fonc.2022.979349

**Published:** 2022-09-08

**Authors:** Meihong Wu, Mao Sheng, Ruomei Li, Xinna Zhang, Xingbiao Chen, Yin Liu, Bin Liu, Yongqiang Yu, Xiaohu Li

**Affiliations:** ^1^ Department of Radiology, The Second People’s Hospital of Hefei, Hefei Hospital Affiliated to Anhui Medical University, Hefei, China; ^2^ Department of Radiology, Research Center of Clinical Medical Imaging, Anhui Province Clinical Image Quality Control Center, The First Affiliated Hospital of Anhui Medical University, Hefei, China; ^3^ Clinical Science, Philips Healthcare, Shanghai, China

**Keywords:** dual-layer dual-energy CT, squamous cell carcinoma, gastroesophageal junction, adenocarcinoma, effective atomic number

## Abstract

**Objective:**

To examine the clinical values of dual-energy CT parameters derived from dual-layer spectral detector CT (SDCT) in the differential diagnosis of squamous cell carcinoma (SCC) and adenocarcinoma (AC) of the gastroesophageal junction (GEJ).

**Methods:**

Totally 66 patients with SCC and AC of the GEJ confirmed by pathological analysis were retrospectively enrolled, and underwent dual-phase contrast-enhancement chest CT with SDCT. Plain CT value, CT attenuation enhancement (△CT), iodine concentration (IC), spectral slope (λ_HU_), effective atomic number (Z_eff_) and 40keV CT value (CT_40keV_) of the lesion in the arterial phase (AP) and venous phase (VP) were assessed. Multivariate logistic regression analysis was performed to evaluate the diagnostic efficacies of different combinations of dual-energy CT parameters. Receiver operating characteristic (ROC) curves were used to analyze the accuracy of dual-energy CT parameters and Delong test was used to compare AUCs.

**Results:**

IC, λ_HU_, Z_eff_ and CT_40keV_ in AP and VP and △CT in VP were significantly higher in the AC group than those in the SCC group (all P<0.05). ROC curve analysis showed that IC, λ_HU_, Z_eff_ and CT_40keV_ in VP had high diagnostic performances, with AUCs of 0.74, 0.74, 0.79 and 0.78, respectively. Logistic regression showed the combination of IC_VP_, λ_HU VP_, CT_40keV VP_ and Z_eff VP_ had the highest AUC (0.84), with a threshold of 0.40, sensitivity and specificity in distinguishing SCC and AC were 93.1% and 73.0%, respectively. Delong test showed that the AUC of △CT_VP_ was lower than other AUCs of dual-energy CT parameters.

**Conclusion:**

Dual-energy CT parameters derived from SDCT provide added value in the differential diagnosis of SCC and AC of the GEJ, especially the combination of IC, λ_HU_, CT_40keV_ and Z_eff_ in VP.

**Advances in knowledge:**

Dual-energy CT parameters derived from dual-layer spectral detector CT provide added value to differentiate AC from SCC at the GEJ, especially the combination of effective atomic number, spectral slope, iodine concentration and 40keV CT value in VP.

## Introduction

In recent years, the incidence of carcinoma of the gastroesophageal junction (GEJ), with the tumor center located within 5cm above or below the gastroesophageal junction and crossing or touching the GEJ, has shown a clear upward trend worldwide (1–3). In comparison to gastric or esophageal cancer, lymph node and hematogenous metastases are more likely to occur from GEJ cancer (4). In addition, the early postoperative recurrence rate is high, and patient prognosis is poor. Recent evidence from the American Joint Committee on Cancer Staging System suggests that SCC of the GEJ shows different clinicopathologic characteristics compared to AC (5). Previous studies (6, 7) have shown mediastinal lymph node metastases are more likely to occur in SCC above the GEJ, whereas metastases from the AC under the GEJ probably appear in the abdomen. It was reported that patient with AC had better prognosis performance than with SCC. The 10-year overall survival rate is >40% and about 20% in AC and SCC, respectively (8). In general, the clinicopathological classification of GEJ cancer is an independent prognostic factor that can affect the surgical pathway and lymph node dissection. So, it is particularly important to differentiate and diagnose SCC and AC accurately at the GEJ.

Relying on the fact that the x-ray attenuation was determined by the material composition, the mass density of the material, and the photon energies, dual-energy CT (DECT) was proposed to provide additional information (9). The main commercially available approaches of dual-energy CT are: rapid tube voltage switching DECT (rsDECT), dual-source DECT (dsDECT) and dual-layer spectral detector CT (SDCT). Many clinical applications and software tools with DECT were developed for use (e.g., isoattenuating gallstones identification, virtual non-calcium maps) (9, 10). SDCT is the latest detector-based dual-energy CT which uses two layers of detectors to separate high- and low energy level x-ray attenuation (9, 10). It can provide both conventional images and several spectral image series from a single acquisition. The spectral image series can provide more quantitative indicators to show microscopic changes in the tumor’s internal tissue structure and hemodynamics. However, few reports have reported these clinical benefits in assessment of GEJ cancer. Zhou Yue et al. (11) reported that the combination of normalized iodine concentration and spectral slope from 40-70 keV in arterial phase (AP) was of great value in differentiating SCC from AC of the GEJ. Ma Yi-Chuan et al. (12) reported that effective atomic number (Z_eff_) in venous phase (VP) had the highest diagnostic efficiency for differentiating SCC and AC of the GEJ. The same rsDECT was used in both studies but with different research outputs.

The purpose of this study was to investigate the clinical values of DECT parameters derived from SDCT in the differential diagnosis of AC and SCC of the GEJ.

## Methods

The institutional review board approved this retrospective study and waived the requirement for written informed consent.

## Patients

The clinical and imaging data of 66 patients with pathologically confirmed AC or SCC of the GEJ in our hospital from February to December 2021 were retrospectively collected. Age, sex, pathological type, degree of differentiation, main clinical symptoms and lesion locations were recorded. Inclusion criteria were: (1) cancer located at the GEJ; (2) AC or SCC of the GEJ confirmed by postoperative pathology or gastroscopic biopsy; (3) spectral enhanced chest CT performed before surgery. Exclusion criteria were: (1) incomplete clinical or imaging data; (2) other related treatments before DECT examination.

## Dual-energy CT examination

All patients underwent SDCT (IQon, Philips Healthcare, Best, the Netherlands), two-phase enhanced chest scan before the treatment. The scan range was from the thoracic inlet to the bottom of the lung. The major scan parameters were: tube voltage, 120 kVp; automatic tube current modulation (tube current-time product 74 mAs to 120 mAs); slice thickness and spacing, 5.0 mm; X-ray tube rotation time, 0.5 s; detector collimation, 64x0.625 mm; pitch, 0.516; field-of-view (FOV), 350mm; reconstruction matrix, 512x512. A high-pressure syringe was utilized to inject 60-80 ml of contrast media (iodixanol, 320 mg iodine/mL) through the cubital vein at a flow rate of 3.0 ml/s. The bolus tracking technology was used, and the arterial and venous phases were started 30 and 60 s after injection, respectively. After scanning, both conventional 120 kVp images and spectral based images (SBIs) were reconstructed. The mean effective radiation dose (ED) of patients is (12.42 ± 2.41) mSv.

## Measurement of quantitative dual-energy CT parameters

All images were transferred to the dedicated post-processing workstation (IntelliSpace Portal, version 10.0; Philips Healthcare). Plain CT value, CT attenuation enhancement (△CT), iodine concentration value (IC), spectral slope (λ_HU_), effective atomic number (Z_eff_) and CT value at the energy level of 40 keV (CT_40keV_) for each lesion in AP and VP were measured. A region of interest (ROI) was placed in the solid component of the lesion in the axial image (with covering >70% in the maximum section, 15-196 mm^2^), and blood vessels, necrotic cystic areas, hemorrhage and calcification areas were avoided as much as possible. All the positions of ROIs delineated on the images of the same lesion at each phase were consistent. Each measurement was repeated three times and averaged. Then, △CT and λ_HU_ were calculated by the following formulae (13):


$$\text{CT attenuation enhancement}:\Delta {\text{CT}}_{\text{AP/VP}}={\text{ CT}}_{\text{AP/VP}}-{\text{CT}}_{\text{Plain scan}}$$

$$\text{Spectral slope}:{\text{ λ}}_{\text{HU}}=({\text{CT}}_{40keV}-{\text{CT}}_{100 keV})/60$$

## Statistical analysis

SPSS 22.0 (SPSS, USA) was used for data analysis. Continuous variables were expressed as mean ± standard deviation (SD). Normally distributed data were compared by the independent samples *t* test between the SCC and AC groups; a non-parametric test was used for non-normally distributed data. The Chi-squared test or the Fisher’s exact probability method was used for comparing categorical data. Multivariate logistic regression analysis was performed for variables with P<0.05 in univariate analysis and predicted probabilities were recorded for further receiver operating characteristic (ROC) curve analysis. ROC curve analysis was performed to evaluate the diagnostic efficacy and Youden index was calculated to determine the optimal diagnostic threshold. Delong test was used to compare AUCs. P<0.05 was considered statistically significant.

## Results

### Patient features

A total of 66 patients (mean age, 74 ± 9 years; 50 men) were enrolled in this study, including 37 AC and 29 SCC cases. Among them, 22 and 44 cases were confirmed by pathology after surgery and gastroscopic biopsy, respectively. There were no significant differences in patient age, gender, tumor location, degree of differentiation and clinical symptoms between the two groups (all P>0.05), as shown in **Table 1**.

**Table 1:** Clinical data of the included patients.

Index	AC (n = 37)	SCC (n = 29)	Z/χ^2^	P
Age^*^, ys	75.51 ± 9.194	72.07 ± 10.351	-1.487	0.137
Sex Male Female	31 (84%) 6 (16%)	19 (66%) 10 (34%)	2.954	0.086
Differentiation degree High Medium to low	5 (14%) 32 (86%)	4 (14%) 25 (86%)	0.000	1.000
Location Above the GEJ Across the GEJ Below the GEJ	10 (27%) 16 (43%) 11 (30%)	7 (24%) 12 (42%) 10 (34%)	0.181	0.913
Symptom Progressive dysphagia Hematemesis or melena Retrosternal or epigastric discomfort	29 (78%) 3 (8%) 5 (14%)	24 (83%) 1 (3%) 4 (14%)	0.628	0.895

A nonparametric test was used for the * group because it did not satisfy the normality criteria; the χ^2^ test or Fisher exact probability method was used in the other groups.

### Imaging manifestation of GEJ cancer

**Figure 1:**
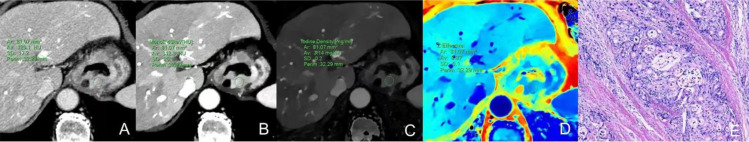
Dual-energy CT findings of venous phase AC of the GEJ. A male patient, 68 years old, was confirmed with AC of the GEJ by postoperative pathology. **(A)** Conventional CT image showing irregular thickening of the wall of the GEJ, luminal stenosis, and obvious enhancement in the venous phase. **(B)** 40_keV_ virtual monochromatic image showing a CT attenuation value of the lesion in the venous phase of 312.3 HU. **(C)** The IC of the solid component of the lesion was3.14 mg/ml. **(D)** The Z_eff_ of the lesion was 8.87. **(E)** Hematoxylin-eosin staining of AC with ×100 magnification.

**Figure 2:**
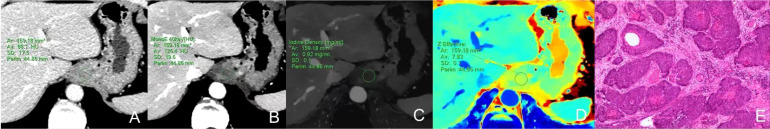
Dual-energy CT findings of venous phase SCC of the GEJ. A male patient, 78 years old, was confirmed with SCC of the GEJ by postoperative pathology. **(A)** Conventional CT image showing irregular thickening of the wall of the GEJ, luminal stenosis, and moderate enhancement in the venous phase. **(B)** 40_keV_ virtual monochromatic image revealed a CT attenuation value of the lesion in the venous phase of 126.4 HU. **(C)** The IC of the solid component of the lesion was 0.92mg/ml. **(D)** The Z_eff_ of the lesion was 7.83. **(E)** Hematoxylin-eosin staining of SCC with ×100 magnification.

Patients with AC or SCC groups had irregular wall thickening at the GEJ, varying degrees of luminal stenosis, and partial formation of soft tissue mass, which showed moderate to obvious uneven enhancement on enhanced CT images (**Figures 1**, **2**). The average diameters of the lesions in AC group and SCC group were about 30.52 ± 10.32mm and 29.61 ± 8.85mm, respectively. Lymph node enlargement was observed in 16 AC and 6 SCC cases from the cardia or lesser curvature of the stomach; 8 AC and 4 SCC cases showed multiple liver metastasis; 3 AC and 8 SCC cases had adrenal metastasis and mediastinal lymph node enlargement, respectively.

### Quantitative dual-energy CT parameters comparison

**Figure 3:**
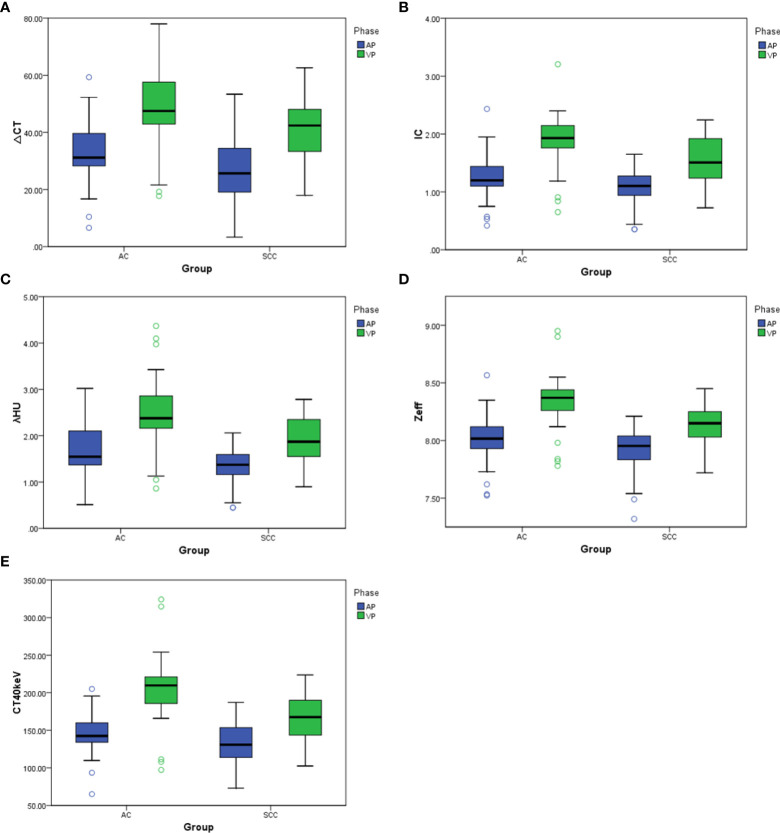
**(A–E)** Box plots of dual-energy CT parameters in AP and VP. ΔCT, IC, λ_HU_, Z_eff_, and CT_40keV_ in AP and VP are higher for AC compared with SCC, to varying degrees, with differences in energy Dual-energy CT parameters greater in VP than in AP.

**Figure 4:**
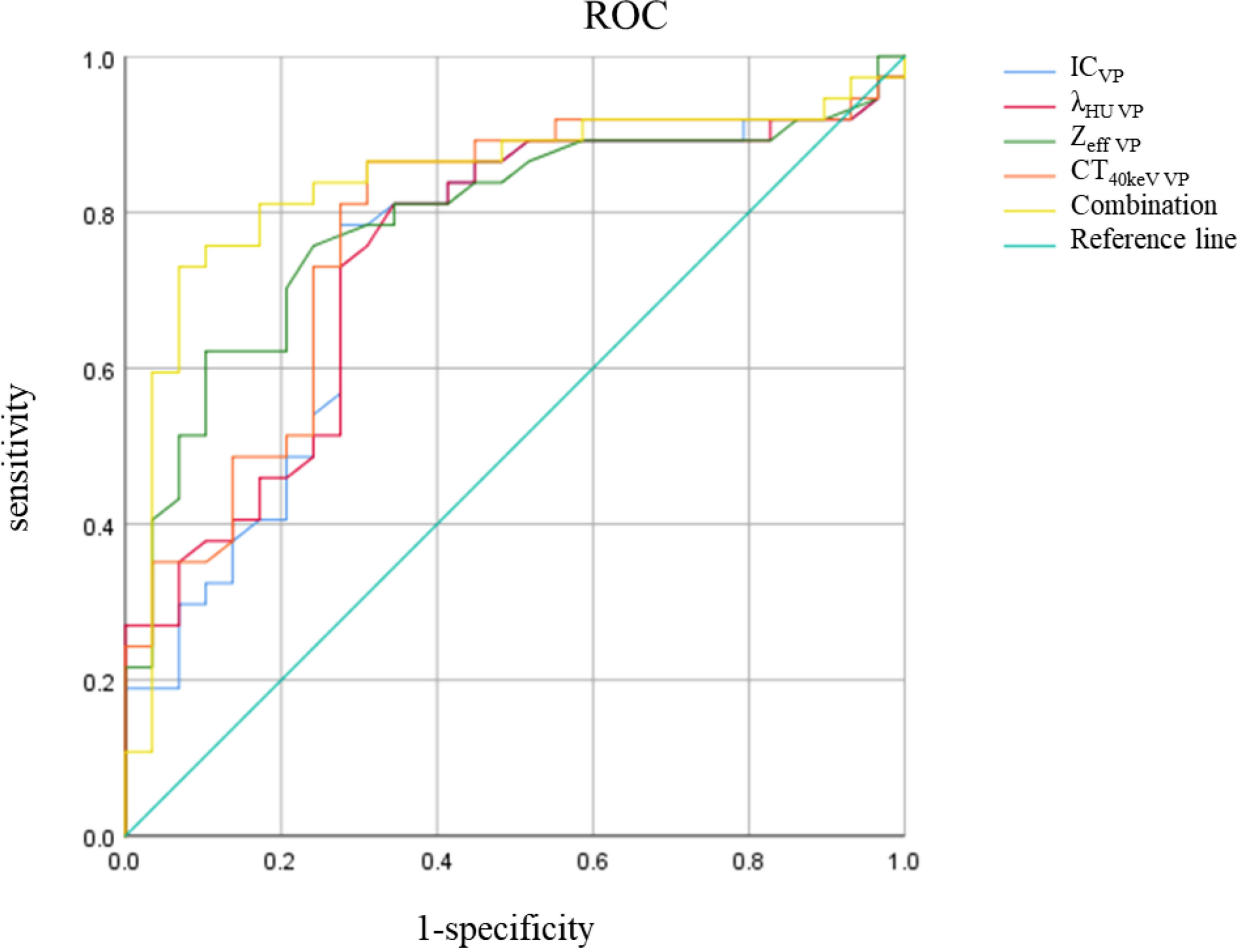
IC, λ_HU_, Z_eff_, CT_40keV_, Combination ROC curve in VP. The combination of IC, λ_HU,_ CT_40keV_ and Z_eff_ in VP had the highest diagnostic efficacy.

There was no significant difference in △CT in AP between the AC and SCC groups (P=0.065). IC, λ_HU_, Z_eff_ and CT_40keV_ in AP and VP, and △CT in VP from AC group were significantly higher than those from SCC group (all P<0.05), as shown in **Table 2** and **Figure 3**. ROC curve analysis showed that the diagnostic efficiencies of IC, λ_HU_, Z_eff_ and CT_40keV_ were higher in VP compared with the AP, with AUCs of 0.74, 0.74, 0.79 and 0.78, respectively. The prediction probabilities from multivariate logistic regression analysis of IC, λ_HU_, Z_eff_ and CT_40keV_ in VP reach the highest AUC (0.84) with a threshold of 0.40, sensitivity and specificity for SCC and AC detection were 93.1% and 73.0%, respectively (**Figure 4**). Detailed results of ROC analysis were listed in **Table 3**. Delong test showed that the AUC of △CT_VP_ was significantly lower than that of combination (IC_VP_, λ_HU VP_, CT_40keV VP_ and Z_eff VP_), P<0.05. 

**Table 2:** Dual-energy CT parameters in the AC and SCC groups in AP and VP.

	AC	SCC		
	Mean (95% CI)	Mean (95% CI)	Difference (95% CI)	P
arterial phase				
△CT	32.08 (28.10-36.06)	26.54 (22.04-31.03)	5.54 (-0.35-11.43)	0.065
IC	1.27 (1.13-1.41)	1.06 (0.92-1.20)	0.21 (0.02-0.41)	0.032
λ_HU_	1.65 (1.46-1.84)	1.31 (1.15-1.48)	0.33 (0.08-0.59)	0.012
Z_eff_	8.01 (7.94-8.09)	7.89 (7.81-7.98)	0.12 (0.008-0.227)	0.037
CT_40keV_	147.64 (135.24-160.04)	129.88 (118.80-140.96)	17.75 (0.96-34.54)	0.039
venous phase				
△CT	49.95 (44.89-55.02)	40.61 (36.19-45.03)	9.35 (2.54-16.16)	0.008
IC	1.96 (1.77-2.15)	1.53 (1.38-1.70)	0.42 (0.17-0.68)	0.002
λ_HU_	2.47 (2.22-2.72)	1.91 (1.72-2.10)	0.56 (0.23-0.88)	0.001
Z_eff_	8.35 (8.26-8.44)	8.13 (8.07-8.20)	0.21 (0.10-0.33)	0.000
CT_40keV_	208.51 (191.97-225.05)	168.66 (155.84-181.48)	39.85 (18.36-61.34)	0.000

△CT, CT attenuation enhancement; IC, iodine concentration; λ_HU_, spectral slope; Z_eff_, effective atomic number; CT_40keV_, CT value at 40 keV. 95% CI, 95% confidence interval.

**Table 3:** Differential diagnostic efficacies of Dual-energy CT parameters in AP and VP.

Index	AUC (95% CI)	Threshold	Sensitivity, %	Specificity, %
IC_AP _(mg/ml)	0.63 (0.49-0.76)	1.11	73.0	51.7
IC_VP_ (mg/ml)	0.74 (0.61-0.86)	1.75	78.4	72.4
λ_HU AP_	0.66 (0.53-0.79)	2.08	27.0	100
λ_HU VP_	0.74 (0.62-0.87)	2.14	81.1	65.5
Z_eff AP_	0.63 (0.49-0.76)	7.80	62.2	62.1
Z_eff VP_	0.79 (0.68-0.90)	8.31	62.2	89.7
CT_40keV AP_ (HU)	0.65 (0.51-0.78)	133.38	75.7	55.2
CT_40keV VP_ (HU)	0.78 (0.66-0.89)	175.75	86.5	69.0
△CT_VP_ (HU)	0.69 (0.56-0.82)	46.3	64.9	69.0
Combination of dual-energy CT parameters*	0.84 (0.74-0.94)	0.40	93.1	73.0

△CT_VP,_ CT attenuation enhancement in the venous phase; IC_AP/VP,_ iodine concentration in the arterial and venous phases; λ_HU AP/VP_, spectral slope in the arterial and venous phases; Z_eff AP/VP,_ effective atomic number in the arterial and venous phases; CT_40keV AP/VP,_ CT value of 40keV in the arterial and venous phases.*dual-energy CT parameters including IC_VP_, λ_HU VP_, CT_40keV VP_ and Z_eff VP_ 95% CI, 95% confidence interval.

## Discussion

Due to the particularities of tumor sites, there are great differences in clinical stages and treatment planning for GEJ tumors. In order to clarify the definition of cancer of the esophagogastric junction and design the therapeutic strategy, Siewert and colleagues published a topographic-anatomic subclassification of adenocarcinomas of the EGJ in 1987 (14). This classification was approved at the consensus meetings of the International Society of Diseases of the Esophagus in 1995 and the International Gastric Cancer Association in 1997 (15). The Siewert classification is purely based on the anatomic localization of the tumor center, which can be defined by endoscopy using the proximal end of the longitudinal gastric mucosa folds as a pragmatic reference for the endoscopic cardia (zero point). Siewert classification of adenocarcinomas of the esophagogastric junction. Siewert I: tumor center 1 cm above to 5 cm above the cardia (zero point). Siewert II: tumor center 1 cm above to 2 cm below the cardia (zero point). Siewert III: tumor center 5 cm below to 2 cm below the cardia (zero point) (14).

In general, Siewert I cancer is epidemiologically and histologically similar to esophageal cancer, which should refer to the clinical staging and surgical strategy of esophageal cancer. While Siewert II and III are epidemiologically and histologically similar to gastric cancer., which should refer to the clinical staging and surgical strategy of gastric cancer (16). Researchers have found that the three Siewert types in Western countries mainly include AC cases, while in Eastern countries Siewert type I mainly comprises SCC cases, and type II and III mainly have AC cases (16, 17). Therefore, better differentiation between SCC and AC at the GEJ would be helpful for clinical stages and treatment plan development. Moreover, a large number of studies have revealed that low-level virtual monochromatic imaging and iodine density fusion imaging can significantly improve the contrast between tumor lesions and surrounding tissues, which enhances lesion visualization and provides added value for clinical staging and Siewert classification of GEJ carcinoma.

The current study showed that dual-energy CT parameters in VP had better differential diagnosis efficiency between SCC and AC than those obtained in AP, and dual-energy CT parameters performed better than conventional CT attenuation enhancement (AUC values of 0.74-0.79 vs 0.69). Interestingly, the combination of IC, λ_HU_, Z_eff_ and CT_40keV_ in VP had the highest AUC (0.84). The possible reason is that CT scan in AP is performed earlier, with insufficient iodine uptake by the lesions, while perfusion in VP is more adequate (12). The diagnostic efficacy in this study was similar to that described by a previous study by Zhou Yue et al. (11) with the highest AUC of 0.804. However, the latter study showed that the AUCs of normalized iodine concentration (NIC), λ_40-70keV_ and Z_eff_ in AP was higher in AC group than those in SCC group, and the combined diagnosis efficiency of NIC and λ_40-70keV_ in AP had the highest AUC (0.804). The main reason for this discrepancy might be the different DECT scanners and scan protocols used. Nevertheless, the previous two studies and ours showed the clinical benefits derived from DECT parameters in differentiating SCC from AC and further studies with large cohorts of patients should performed.

The iodine density map in Dual-energy CT mainly reflects the blood supply status of the lesion; the higher the IC value, the richer the lesion’s blood supply (18). The above results showed that the IC values of AC of the GEJ in AP and VP were higher than those of SCC, indicating that AC has higher vascularity than SCC. The possible reason is that AC grows diffusely in the wall, and the internal pressure of the tissue is small, which promotes growth and opening of new blood vessels, while SCC shows continuous-accumulation growth, which does not promote the growth and development of new blood vessels (19). The current results corroborated a study on lung AC and SCC (20). Effective atomic number reflects the material’s composition, which can be used for lesion detection or differentiation (21). In this study, Z_eff_ in VP had the highest diagnostic performance, with an AUC of 0.79 in differentiating AC and SCC, corroborating low-energy level (e.g., 40keV) virtual monochromatic imaging can significantly improve the CT attenuation of lesions containing a contrast agent, which is helpful for lesion detection, tumor delineation and artery assessment (21). As shown above, the CT_40keV_ values of AC in AP and VP were higher than those of SCC, also resulting in a good AUC of 0.78 in VP.

This study had some limitations. First, the sample size was relatively small. Secondly, metastatic lymph nodes were not further analyzed. Thirdly, the microvessels of tumors were not confirmed, and correlations with DECT parameters were not analyzed.

In conclusion, dual-energy CT parameters derived from dual-layer spectral detector CT provide added value to differentiate AC from SCC at the GEJ, especially the combination of effective atomic number, spectral slope, iodine concentration and 40-keV CT value in VP.

## Data availability statement

The original contributions presented in the study are included in the article/supplementary material. Further inquiries can be directed to the corresponding author.

## Ethics statement

The studies involving human participants were reviewed and approved by The Second People’s Hospital of Hefei, Hefei Hospital Affiliated to Anhui Medical University review board approved this retrospective study and waived the requirement for written informed consent. The ethics committee waived the requirement of written informed consent for participation.

## Author contributions

All authors listed have made a substantial, direct, and intellectual contribution to the work and approved it for publication.

## Conflict of interest

Author XC was employed by Philips Healthcare.

The remaining authors declare that the research was conducted in the absence of any commercial or financial relationships that could be construed as a potential conflict of interest.

## Publisher’s note

All claims expressed in this article are solely those of the authors and do not necessarily represent those of their affiliated organizations, or those of the publisher, the editors and the reviewers. Any product that may be evaluated in this article, or claim that may be made by its manufacturer, is not guaranteed or endorsed by the publisher.

## Abbreviations

GEJ, gastroesophageal junction; SDCT, Dual-layer spectral detector CT; SCC, squamous cell carcinoma; AC, adenocarcinoma.
